# Study of the Stability and Anti-Inflammatory Activity of Paeonol–Oleanolic Acid Liposomes by Microfluidic Technology

**DOI:** 10.3390/foods14122030

**Published:** 2025-06-08

**Authors:** Xianzheng Ma, Hui Zhang, Jinkai Luan, Mingfa Tian, Xiuxin Zhang, Ammara Sohail, Dong Liang, Jiguo Liu, Fuzhan Tao, Zheng Wang, Daijie Wang

**Affiliations:** 1 College of Pharmacy, Food Resources Development and Health Product Creation International Joint Laboratory/Biological Engineering Technology Innovation Center of Shandong Province, Heze Branch of Qilu University of Technology, Shandong Academy of Sciences, Heze 274000, China; 15106912350@163.com (X.M.); zhcharmingqueen@163.com (H.Z.); kari323682567@163.com (J.L.); t15165141920@163.com (M.T.); sohailammara9@gmail.com (A.S.); 2 Institute of Vegetables and Flowers, Chinese Academy of Agricultural Science, Beijing 100081, China; zhangxiuxin@caas.cn; 3 Department of Chemistry, University of Okara, Okara 56300, Pakistan; 4 Heze Peony Industrial Technology Research Institute, Heze 274000, China; szfbgsmdb@163.com (D.L.); hzljg919@163.com (J.L.); hzstfz@126.com (F.T.); 5 Department of Genetics and Cell Biology, Basic Medical College, Qingdao University, Qingdao 266071, China; zheng.wang@qdu.edu.cn

**Keywords:** liposome, paeonol, oleanolic acid, cholesterol, anti-inflammatory activity

## Abstract

(1) Background: This study used a microfluidic approach to prepare paeonol (PAE) liposomes with oleanolic acid (PAE-ONLs) instead of cholesterol (PAE-CNLs), aiming to reduce cholesterol levels and enhance stability and anti-inflammatory activity. (2) Methods: The liposome formula was optimized, characterized, and tested for anti-inflammatory activities in zebrafish and RAW 264.7 macrophages, utilizing various stability and molecular interaction methods. (3) Results: The best PAE-ONL preparation conditions were 10.25 mg/mL of soy lecithin, 0.82 mg/mL of oleanolic acid, and 0.22% (wt%) of Tween 80, with an *EE* of 64.61 ± 0.42%. TEM confirmed the uniform spherical morphology, and FTIR confirmed that oleanolic acid was incorporated into the liposomes. PAE-ONLs showed better stabilities than PAE-CNLs. Molecular interaction results revealed that PAE-ONLs achieved a greater energy reduction, reaching −85.07 kJ/mol vs. the −62.64 kJ/mol of PAE-CNLs, with stable hydrogen bonding interactions. PAE-ONLs significantly reduced inflammatory cell migration in zebrafish and decreased NO, TNF-α, IL-6, and IL-1β levels in LPS-induced RAW 264.7 macrophages at 20 μg/mL. A network pharmacology analysis showed that oleanolic acid and paeonol interacted with 45 and 11 anti-inflammatory targets, respectively, and their combination in PAE-ONLs enhanced their anti-inflammatory coverage. (4) Conclusions: PAE-ONLs, utilizing oleanolic acid as a cholesterol substitute, exhibit enhanced stability and superior anti-inflammatory effects.

## 1. Introduction

Liposomes are frequently used as drug delivery vehicles because they can encapsulate hydrophobic bioactive substances [1]. Liposomes are spherical vesicles that have one or more concentric phospholipid bilayers around an aqueous center [2]. Because liposomes are biodegradable and harmless, they offer an effective way to administer several medications [3]. Along with phospholipids, cholesterol is a necessary component of liposomes and is frequently utilized as a membrane material for encapsulating medications [4]. However, consuming too much cholesterol increases the risk of hyperlipidemia and cardiovascular diseases [5]. Furthermore, cholesterol has a significant impact on their surface and physical characteristics [6]. Therefore, while producing liposomes, it is vital to look for alternatives to cholesterol.

Oleanolic acid (OA), as a pentacyclic triterpenoid, has a broad spectrum of pharmacological activities, including antiviral, antitumor, anti-inflammatory, and anticancer effects [7,8,9,10]. OA’s biological activities are attributed to its hydroxyl groups at the C3 position and the pentacyclic ring system, which influence membrane fluidity and stability, contributing to its pharmacological potential [11]. It has the same polycyclic rigid planar structure and 3β-hydroxyl group as cholesterol, and it has been found to enhance the physicochemical properties of phospholipid bilayers and alter the stability and fluidity of liposome membranes [12]. Therefore, OA was chosen as a cholesterol substitute for liposomal stabilization due to its structural similarities, recognized biological actions, and feasibility in stabilizing liposomes.

Paeonol (PAE), with a long history of clinical applications in China, possesses numerous pharmacological activities, including anti-inflammatory, antibacterial, anti-allergic, and antitumor activities [13,14,15,16]. Among them, the anti-inflammatory effect is particularly prominent [17]. Despite its promise in traditional medicine, its medicinal applications are restricted due to its low absorption and stability [18]. Liposomal delivery technology tries to solve this problem by enhancing PAE delivery and stability. This might greatly increase its clinical value, addressing a key therapeutic need for safe, effective anti-inflammatory drugs with multi-targeted effects. Studies have indicated that encapsulating PAE in liposomes may greatly improve its stability and water solubility [19]. To date, only a few studies have documented the production of oleanolic acid-based liposomes for the delivery of PAE.

Traditional liposome production methods, such as membrane extrusion, ethanol injection, micro-emulsification, and thin-film hydration, depend on bulk-scale synthesis and usually produce a narrow range of size and homogeneity [20]. These macroscale methods may result in batch-to-batch variations in the important quality metrics of the average particle size and polydispersity index (PDI) [21]. For liposome preparation, more recent microfluidic-based technologies can reduce scale-up issues and improve manufacturing efficiency, automation, and time [22]. The PDI and average particle size are two important liposome quality parameters that can be better controlled using microfluidic-based preparation [23].

In this work, oleanolic acid was used to substitute cholesterol to prepare liposomes (ONLs). Single factors and the RSM method were used to develop and improve the preparation of paeonol liposomes with oleanolic acid (PAE-ONLs) and cholesterol (PAE-CNLs). Fourier transform infrared spectroscopy (FTIR) and transmission electron microscopy (TEM) were used to characterize the liposomes. Their particle size, zeta potential, and encapsulation efficiency (*EE*) were evaluated. Stability tests were conducted, while molecular interactions were examined. Zebrafish and LPS-induced RAW 264.7 macrophages were employed to evaluate their anti-inflammatory effects. Network pharmacology methods were applied to investigate the synergistic anti-inflammatory effects of PAE and OA in PAE-ONLs through an analysis of their overlapping targets and pathways, coupled with molecular docking to examine their binding interactions. By substituting oleanolic acid for cholesterol, this study created a novel kind of liposome that outperformed traditional liposomes that included cholesterol. This liposome’s simplicity, adaptability, effectiveness, and safety make it a promising candidate for clinical translation.

## 2. Materials and Methods

### 2.1. Materials and Chemicals

RAW 264.7 macrophages were acquired from Procell Science & Technology Co., Ltd., Wuhan, China. Oleanolic acid and paeonol were bought from Desite Biological Technology Co., Ltd. (Chengdu, China), with purities over 98%. The suppliers of soy lecithin, mannitol, and cholesterol were Macklin Biochemical Technology Co., Ltd. in Beijing, China. We bought Griess reagent from Thermo Fisher Scientific in Cleveland, OH, USA. The supplier of LPS (*Escherichia coli* 0127: B8) was Sigma Chemical Co., Ltd. (St. Louis, MO, USA). Procell Company, Wuhan, China, supplied the Dulbecco’s Modified Eagle Medium (DMEM) and fetal bovine serum (FBS). A Cell Counting Kit-8, or CCK-8, was acquired from Beyotime Biotechnology, Shanghai, China. ELISA kits for interleukin-6 (IL-6; batch WWWW03J6865283), interleukin-1β (IL-1β; batch WWWW044JHN8410), and tumor necrosis factor-alpha (TNF-α; batch WWWW06ZR4L1380) were acquired from Elabscience Biotechnology (Wuhan, China). A dialysis bag (Mr. 7000) was acquired from Solarbio Science & Technology Co., Ltd. (Beijing, China). A microfluidic chip was acquired from Zhongxin Qiheng Scientific Instrument Co., Ltd., Suzhou, China. A constant-flow pump for the microfluidic chip was acquired from Baoding Precision Pump Co., Ltd. (Baoding, China). HPLC-grade ethanol was acquired from China National Medicines Corporation Ltd. (Shanghai, China). Phosphate-buffered saline (PBS) at a pH of 6.8 was purchased from Procell Life Science Co., Ltd. (Beijing, China).

### 2.2. Optimizing the Preparation Conditions of Liposomes

#### 2.2.1. Preparation of Liposomes

**Figure 1:**
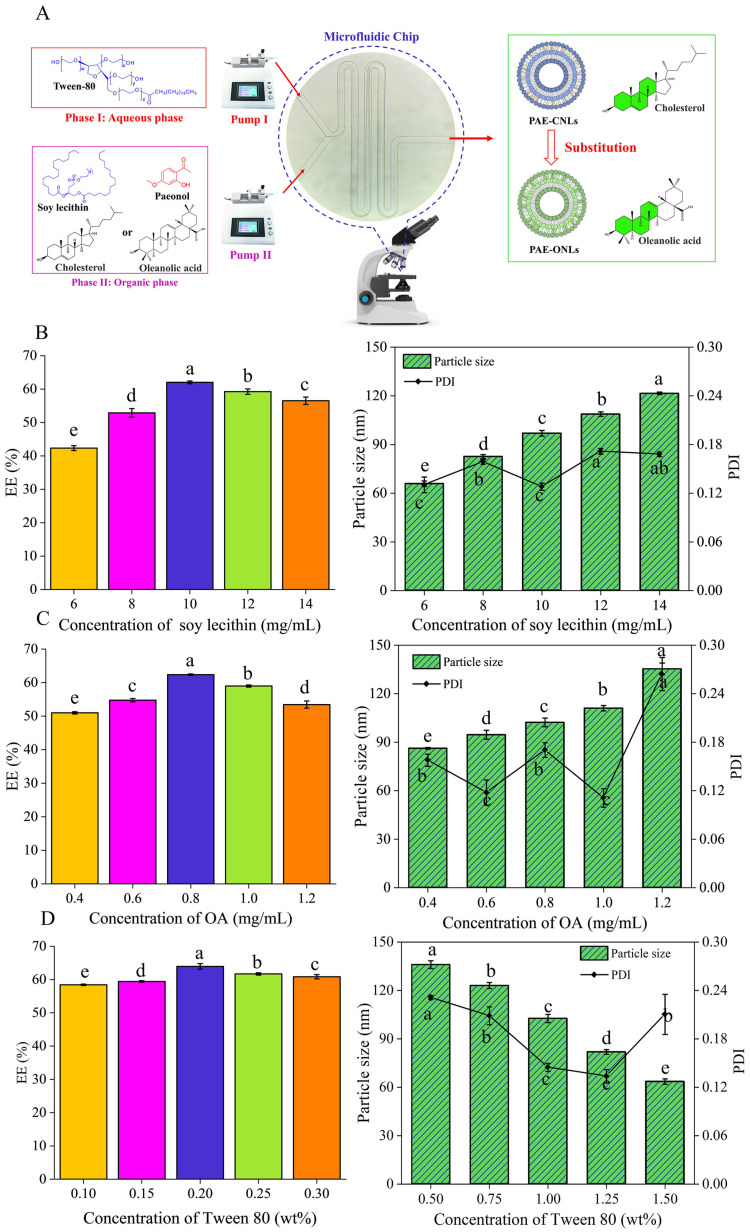
Microfluidic preparation of PAE-ONLs and optimization of preparation parameters through single-factor experiments. (**A**) Schematic diagram showing the microfluidic chip design and liposome formation process. Effects of (**B**) soy lecithin concentration (mg/mL), (**C**) oleanolic acid concentration (mg/mL), and (**D**) Tween 80 concentration (wt%) on the *EE*, particle size, and PDI. The various letters indicate significant differences (*p* < 0.05) across samples, and the data show mean ± standard deviation (*n* = 3).

Liposomes were manufactured by combining the organic and aqueous phases at a certain flow rate ratio using microfluidic technology. Tween 80 and ultrapure water made up the aqueous phase. Soy lecithin, paeonol, and cholesterol or oleanolic acid dissolved in ethanol in a certain weight made up the organic phase. The aqueous and organic phases were injected into the first and second inlets to achieve unbalanced splitting and cross-collision (Figure 1). For further examination, all of the liposome solutions were kept at 4 °C.

#### 2.2.2. Paeonol Encapsulation Efficiency

First, 2 mL of liposome solution was added to a dialysis bag with a molecular weight cut-off of 7 kDa and left in PBS (pH 6.8, 0.2 M) for six hours. The total quantity of the active component was evaluated by ultrasonically emulsifying the liposomal emulsion with methanol and then centrifuging (40 min, 4000 r/min) to remove any insoluble debris. The resultant solution was analyzed using high-performance liquid chromatography (HPLC) to measure the total PAE concentration. The HPLC analysis for paeonol was conducted using a Wooking-K2025 HPLC system from Wooking Instrument Co., Ltd., Shanghai, China. A Thermo Scientific C_18_ column (250 × 4.0 mm, 5 µm) was employed in the HPLC analysis. The HPLC conditions for paeonol were methanol and water (60:40, *v*/*v*) as mobile phases; a flow rate of 0.8 mL/min; wavelength detection at 274 nm; a sample injection volume of 20 μL; and a column temperature maintained at 30 °C. The HPLC conditions for oleanolic acid were methanol and formic acid solution (90:10, *v*/*v*) as mobile phases; a flow rate of 1.0 mL/min; wavelength detection at 210 nm; a sample injection volume of 20 μL; and a column temperature maintained at 30 °C. The following formula was used to determine the *EE* of paeonol:

$$\mathrm{Paeonol}\;\mathrm{encapsulation}\;\mathrm{efficiency}\;(\%)=\frac{\mathrm{Loaded}\;\mathrm{amount}\;\mathrm{of}\;\mathrm{paeonol}\;({PAE}_{L})}{\mathrm{Total}\;\mathrm{amount}\;\mathrm{of}\;\mathrm{paeonol}\;({PAE}_{T})}\times 100\%$$

where *PAE_L_* refers to the quantity encapsulated in liposomes, while *PAE_T_* represents the total PAE added during liposome preparation.

#### 2.2.3. Experimental Design for Optimization of PAE-ONLs

To identify important parameters influencing particle size and encapsulation performance, single-factor studies of the PAE-ONL preparations were conducted. A range of liposomes was created by varying the amounts of soy lecithin, OA, and Tween 80. A three-factor, three-level Box–Behnken design (BBD) response surface approach was used to identify the ideal circumstances based on the single-factor trials to investigate the interactions between various components. The response value and the chosen process components were connected using the second-order nonlinear quadratic polynomial model. Table S1 describes the experimental variables’ encoding table. To improve the preparation process, the BBD created a total of 17 experimental groups, which were subsequently examined (Table S2). Design-Expert 13.0.1.0 software (Stat-Ease Inc., Saint Paul, MN, USA) was used to analyze the data.

### 2.3. Characterization

#### 2.3.1. Particle Size Distribution and Zeta Potential

The dynamic light scattering (DLS) method was used to evaluate the average size and PDI, while microelectrophoresis was used to measure the zeta potential with a Malvern Zetasizer Nano ZS90 (Malvern Instruments, Malvern, Worcestershire, UK). The refractive index was 1.420, representing a weighted average RI of the liposomal system, which accounts for the phospholipid bilayer and aqueous core, is aligned with standard practices for analyzing liposomes with a low or unloaded drug content, and is consistent with the default recommendations provided by DLS instrument software. The equipment automatically determined the average size, which was the mean of the intensity size [24]. Every sample was measured three times.

#### 2.3.2. TEM Analysis

TEM was used to analyze the liposomes’ morphology and form. On a copper grid covered with Formvar carbon (200 mesh), a single drop of the diluted solution (1:500, *v*/*v*) was applied and allowed to air dry at room temperature. After that, 2% phosphotungstic acid was used to stain the samples, and filter paper was used to remove any extra liquid [25]. After being allowed to air dry at ambient temperature, each sample solution was inspected under an FEI Talos F200s microscope (Eindhoven, The Netherlands).

#### 2.3.3. FTIR Analysis

Mannitol was added to the liposomes as a lyoprotectant, and the mixtures were then precooled at −80 °C for a whole day. Through the process of freeze-drying, the samples were reduced to powder. Then, 20 mg of the sample powder was mixed with KBr (1:100, *w*/*w*). The sample was compacted into pellets and subjected to 32 scans at 4000–400 cm^−1^ using an FTIR spectrometer (Bruker Corporation, Karlsruhe, Germany).

### 2.4. Stability Studies

#### 2.4.1. Storage Stability

The storage stability was examined in a dark environment for 30 days. Particle size and paeonol retention were assessed by collecting samples at 0, 3, 6, 9, 12, 15, and 30 days, which were kept at two distinct temperatures of 4 °C and 25 °C. The average size was detected by the DLS technique. Sample solutions of 2 mL with PAE-CNLs and PAE-ONLs were loaded in dialysis bags and placed into 200 mL of ultrapure water for dialysis for 6 h. After dialysis, 6 mL of methanol was added to the liposome solution (approximately 2.5 mL) for ultrasonication. Subsequently, the solution was centrifuged at 10,000 r/min for 30 min. The supernatant was detected by HPLC. The paeonol retention was calculated by dividing the undestroyed liposomes by the total paeonol.

#### 2.4.2. Thermal Stability

The thermal stability was examined at different temperatures, namely, 45 °C, 65 °C, and 85 °C, by adding PAE-CNL and PAE-ONL solutions. The average size was detected by the DLS technique. After 60 min of pretreatment, sample solutions of 2 mL with PAE-CNLs and PAE-ONLs were loaded in dialysis bags and placed into 200 mL ultrapure water for dialysis for 6 h. After dialysis, 6 mL of methanol was added to the liposome solution (approximately 2.5 mL) to perform ultrasonication. The paeonol retention was detected using the same method described above for storage stability.

#### 2.4.3. Salt Stability

Based on previous stability studies, the salt stability was optimized and adjusted in this work [26]. The PAE-CNL and PAE-ONL samples were incubated in NaCl solutions with different concentrations (0, 50, 100, 200, and 400 mM) at 25 °C for 1 h to assess the ionic stability of the liposomes. DLS was used to evaluate the changes in the particle size, PDI, and zeta potential during incubation. By comparing the changes in these parameters throughout the measured ionic strengths, the impact of varying salt concentrations on the stability of the liposomal formulations was examined.

#### 2.4.4. pH Stability

To evaluate the pH stability of the liposomes, the dispersions of PAE-CNLs and PAE-ONLs were adjusted to pH values of 2.0, 7.0, 9.0, and 11.0 using HCl or/NaOH (0.1 M). The samples were incubated at room temperature for 30 min, as previously described in [27], with slight modifications. DLS was used to assess the particle size and zeta potential following incubation. The pH stability of the liposomal formulations was evaluated by analyzing the changes in these parameters over the range of pH values.

### 2.5. Comparison of Thermodynamic Stability and Hydrogen Bonding

To assess the stability of the liposomes composed of oleanolic acid and cholesterol, Density Functional Theory (DFT) calculations were conducted using the Gaussian 16 software package. The B3LYP hybrid exchange–correlation functional with the 6–31 G (d) basis set was employed for geometry optimization and frequency calculations. All optimized geometries were confirmed to be global minima by the absence of imaginary frequencies. The SMD solvation model was used to simulate solvation effects, with water as the solvent and a dielectric constant of ε = 78.36. The Multiwfn 3.6 program was utilized for a wavefunction analysis and electrostatic potential fitting, while VMD 1.9.3 was used for visualizing electrostatic potential maps [28]. The following particular formulae can be used to determine and break down the interaction energy between various molecular segments:

ΔE = E*_AB_* − E*_A_* − E*_B_* + E*_BSSE_* (1)

ΔE*_total_* = ΔE*_elst_* + ΔE*_exch_* +ΔE*_ind_* + ΔE*_disp_* (2)

where E*_AB_*, E*_A_*, and E*_B_* in Equation (1) are the single energy of the binary complex, isolated *A*, and isolated *B*, respectively, and E*_BSSE_* is the correction of the basis set superposition error [29]. ΔE*_elst_*, ΔE*_exch_*, ΔE*_disp,_* and ΔE*_ind_* in Equation (2) are the electrostatic, exchange, dispersion, and induction terms, respectively.

### 2.6. Evaluation of Anti-Inflammatory Activity in Zebrafish

#### 2.6.1. Feeding and Ovulation of Zebrafish

The transgenic zebrafish strain *Tg* (*Lyz: EGFP JS7*) was used, provided by the Zebrafish Drug Screening Platform (Institute of Biotechnology, Shandong Academy of Sciences). The research was carried out in compliance with the National Institutes of Health regulations (Protocol No. SWS20230615, approval date: 15 June 2023, Publication No. 8023, revised in 1996), with approval from the Animal Ethics Committee of the Biology Institute of Shandong Academy of Sciences.

The transgenic zebrafish *Tg* (*Lyz: EGFP JS7*) were raised at 28.5 °C, with a daily light/dark cycle of 14 h and 10 h. All zebrafish were fed normally for two weeks (i.e., zebrafish that did not ovulate within that time frame). The male and female zebrafish were kept apart in a 1:1 ratio in a fish tank. The female and male zebrafish were divided by a partition board for the night, and the partition plate was removed the following morning. Additionally, the male and female zebrafish copulated to lay eggs, and, after three hours, the eggs were collected. All of the eggs were cultured in E3 fish water, which contained 0.33 mM MgSO_4_, 5 mM NaCl, 0.33 mM CaCl_2_, and 0.17 mM KCl. After 10 h, 0.003% phenylthiourea (PTU) was added to the cells to prevent the development of black spots in the zebrafish.

#### 2.6.2. Anti-Inflammatory Activity of Liposomes

Normal zebrafish larvae were picked using a stereo microscope and transferred into a 24-well culture plate after the embryos reached 3 dpf. The zebrafish were divided into a control group (embryonic culture water), a blank group, and various conc. drug treatment groups (each consisting of 10 zebrafish). In the meantime, 2.0 mL of culture water was introduced to two double holes that had been prepared. The negative control group and test group of zebrafish were housed in a light incubator at 28 °C to allow the embryos to continue growing. After 5 h, the zebrafish in the control and blank groups were fed their usual food, whereas the drug treatment groups (PAE-CNLs and PAE-ONLs) received medications at levels of 5, 10, and 20 μg/mL. After 2 h, 20 μM CuSO_4_ solution was added to both the drug and control groups. The zebrafish were left in the dark for 1 h before being cleaned and given an anesthetic. We counted and photographed the number of inflammatory cells that were moving to the zebrafish’s lateral line.

### 2.7. Inflammation in RAW 264.7 Macrophages Stimulated by LPS

#### 2.7.1. Cell Culture

DMEM with 10% heat-inactivated FBS and 1% penicillin–streptomycin was used to cultivate RAW 264.7 macrophages. The cells were kept in a humidified incubator with 5% CO_2_ at 37 °C [30].

#### 2.7.2. Measurement of Cell Viability

The effect of PAE on cellular activity was evaluated using a CCK_8_ assay [31]. Normal cultures of RAW 264.7 cells were planted in 96-well plates with 100 µL of cell suspension per well, passaged more than three times, counted, adjusted to a cell density of 1 × 10^5^ cells/mL, and then incubated for 20 h at 37 °C. The paeonol solution was created with gradient concentrations of 12.5, 25, and 50 µg/mL, coupled with paeonol concentrations of 12.5, 25, and 50 µg/mL. Following the culture, the cell supernatant was disposed of, while 100 µL of the paeonol concentration gradient solution was poured into each well. The cell supernatant was disposed of, and 100 µL of 10% CCK_8_ DMEM solution was poured into each well after removing the plate. The plate was then placed back in the 37 °C incubator for an additional hour of culture. A microplate reader was used to measure the OD at 450 nm, record the data, and calculate the relative cell viability.

#### 2.7.3. Determination of the Concentrations of Inflammatory Factors

A suspension of RAW 264.7 cells (1 × 10^5^ cells/mL) was applied to 96-well plates for 12 h at 37 °C in an incubator with 5% CO_2_. PAE, PAE-CNLs, and PAE-ONLs at several concentrations (2.5, 5, 12.5, and 20 μg/mL) and 1.5 μg/mL LPS were added to the supernatant and incubated for 24 h. The absorbance at 540 nm was measured after mixing 100 μL of culture supernatant with 100 μL of 10% Griess reagent (1:1 *v*/*v*). As a benchmark to evaluate nitrite production, sodium nitrite (NaNO_2_) was utilized to calculate the NO production. The contents of TNF-α, IL-6, and IL-1β were determined in the culture medium supernatants with commercially available ELISA kits (Exp. Date: 24 June 2025), according to the manufacturer’s protocols.

### 2.8. Synergistic Anti-Inflammatory Effects by Network Pharmacology-Based Investigation

To investigate the synergistic anti-inflammatory effects of PAE, OA, and their combination in PAE-ONLs, network pharmacology methods were applied. Drug targets for both PAE and OA were predicted using the Swiss Target Prediction database. Anti-inflammatory genes were obtained from the GeneCards and OMIM databases. A Compound–Target–Disease network was constructed using Cytoscape 3.9.1 to analyze the interactions between the compounds and their targets. Pathway enrichment was carried out through a KEGG analysis via the DAVID database, identifying critical inflammation-related pathways. The overlapping targets of PAE, OA, and PAE-ONLs were examined, which suggested that the combination of both compounds could enhance the anti-inflammatory effect by regulating multiple inflammation-related pathways simultaneously, providing an advantage over using each compound alone. Subsequently, MOE software was employed for molecular docking to examine the interaction and binding affinity of PAE and OA with the hub targets identified in the protein–protein interaction network.

### 2.9. Statistical Analyses

The mean ± standard deviation (SD) was used to characterize the results. An ANOVA with Tukey’s post hoc test in Design-Expert 13 was used to identify significant differences (*p* < 0.05). IBM SPSS 23.0 was employed for statistical analysis, and Duncan’s test (*p* < 0.05) was used to determine whether the differences were significant.

## 3. Results

### 3.1. Single-Factor Experiments

#### 3.1.1. Effect of Soybean Phosphatide Concentration on the *EE* of PAE-ONLs

The concentrations of phospholipids were tested at 6, 8, 10, 12, and 14 mg/mL while maintaining oleanolic acid at 1 mg/mL and Tween 80 at 0.25%. The *EE* increased from 42.35% to 61.35% as the phospholipid concentration increased from 6 to 10 mg/mL, and it decreased to 56.51% at 14 mg/mL (Figure 1B). The PDI remained relatively stable across concentrations (Figure 1B), suggesting that 10 mg/mL was the optimal phospholipid concentration.

#### 3.1.2. Effect of Oleanolic Acid Concentrations on the *EE* of PAE-ONLs

With soy lecithin fixed at 10 mg/mL and Tween 80 fixed at 0.25%, oleanolic acid concentrations were varied from 0.4 to 1.2 mg/mL to examine their effects on the *EE* of PAE-ONLs. The *EE* increased from 44.32% to 63.71% between 0.4 and 0.8 mg/mL, and then it decreased to 41.32% at higher concentrations (Figure 1C). Particle size increased from 86.2 nm to 135.36 nm with an increasing oleanolic acid concentration. The PDI remained below 0.3, even after rising to 0.254 at the critical threshold (Figure 1C), indicating good stability and homogeneity.

#### 3.1.3. Effect of Tween 80 Concentrations on the *EE* of PAE-ONLs

Using optimal soy lecithin (10 mg/mL) and oleanolic acid (0.8 mg/mL) concentrations, Tween 80 was varied from 0.1 to 0.3 wt%. The *EE* increased from 52.09% to 63.96% between 0.1 and 0.2 wt%, and then it decreased to 55.18% (Figure 1D). Particle size decreased from 136.03 nm to 63.52 nm at 0.3 wt% Tween 80, while the PDI remained stable around 0.21 (Figure 1D). It can be assumed that Tween 80, as a stabilizing surfactant, promotes membrane fluidity and consistent nanocarrier production through microfluidic methods, improving drug encapsulation efficiency and liposome stability.

### 3.2. Optimization of PAE-ONLs by the Theoretical Response Surface Models

Table S2 shows the combined effect of the three factors (soy lecithin concentration, oleanolic acid concentration, and Tween 80 concentration) on EE at three levels, with 17 BBDs optimizing the parameters. The fitted equations for estimating the maximum efficiency are provided below:

*Y* = 64.19 + 1.27*A* + 0.7531*B* +0.9938*C* − 1.36*AB* + 0.7750*AC* + 1.15*BC* − 5.16*A*^2^ − 3.43*B*^2^ –2.73*C*^2^, (*p* < 0.05) (3)

where *Y* represents the encapsulation efficiency (*EE*), *A* is the conc. of soy lecithin, *B* is the conc. of oleanolic acid, and *C* is the conc. of Tween 80.

As shown in Table S3, the analysis of variance (ANOVA) demonstrated that the model was highly significant (*F*-value = 79.54, *p* < 0.01). The high coefficient of determination (*R*^2^ = 0.9903) indicated that the established model was adequate for predicting the optimal formulation conditions.

**Figure 2:**
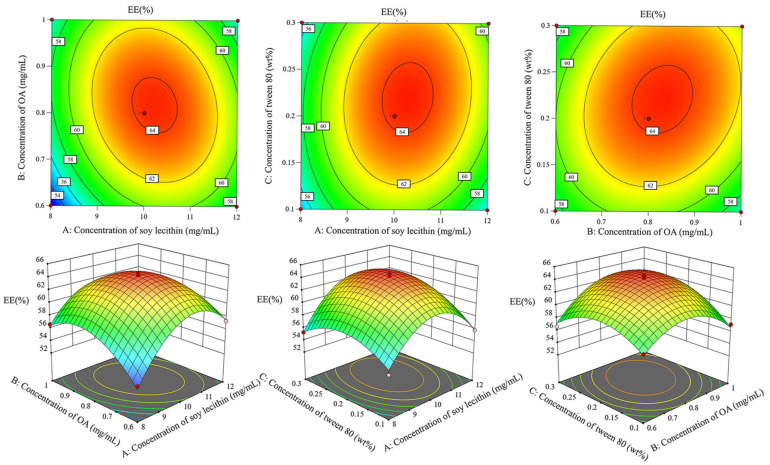
Two-dimensional contour plots and three-dimensional response surface plots illustrate the different factors’ effect on *EE*.

The 3D response surface plots and 2D contour plots in Figure 2 visualize the interactions between variables and their effects on *EE*. The plots revealed that all three factors exhibited significant quadratic effects on *EE*. The interaction between soy lecithin and oleanolic acid concentrations showed that *EE* initially increased with higher concentrations of both components but decreased after reaching optimal levels. Similar patterns were observed for the interactions between soy lecithin and Tween 80, as well as between oleanolic acid and Tween 80.

Through numerical optimization, the optimal preparation conditions were determined to be a soy lecithin concentration of 10.25 mg/mL, an oleanolic acid concentration of 0.82 mg/mL, and a Tween 80 concentration of 0.22%. Under these optimized conditions, the experimental *EE* value (64.61 ± 0.42%) validated the accuracy of the response surface methodology.

### 3.3. Characterization

**Figure 3:**
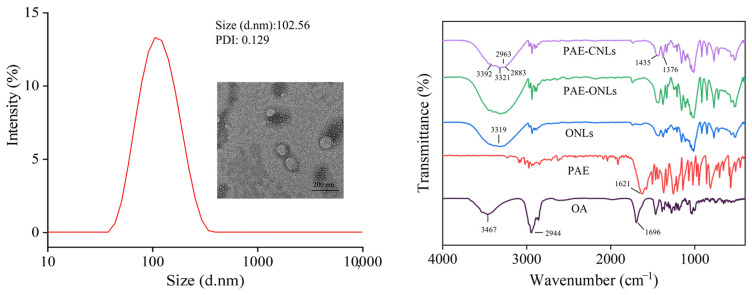
Characterization of PAE-ONLs. Particle size distribution curve with TEM image. FTIR spectra of OA, PAE, ONLs, PAE-ONLs, and PAE-CNLs.

The PAE-ONLs prepared under optimized conditions showed a uniform size distribution, with an average particle size of 102.65 ± 2.55 nm and a PDI of 0.129 ± 0.015. The zeta potential value was −13.3 ± 0.833 mV, demonstrating a moderate formulation stability. TEM imaging revealed that PAE-ONLs were spherical and dispersed uniformly in the suspension (Figure 3A).

An FTIR analysis revealed distinct spectral changes after liposome formation (Figure 3B). The characteristic peaks of pure paeonol (1621 cm⁻^1^) and oleanolic acid (1696 cm⁻^1^) significantly weakened or disappeared in the PAE-ONL spectrum. A notable shift was observed in the OH stretching band from 3467 cm⁻^1^ (pure oleanolic acid) to 3319 cm⁻^1^ (PAE-ONLs), which verified that hydrogen bonding formed between paeonol and oleanolic acid. Additionally, the strong bands at 2944 cm⁻^1^ and 1696 cm⁻^1^ became significantly weaker in the liposome spectrum. These results show that oleanolic acid and paeonol were successfully integrated into the liposome matrix.

### 3.4. Stability Studies

#### 3.4.1. Storage Stability

**Figure 4:**
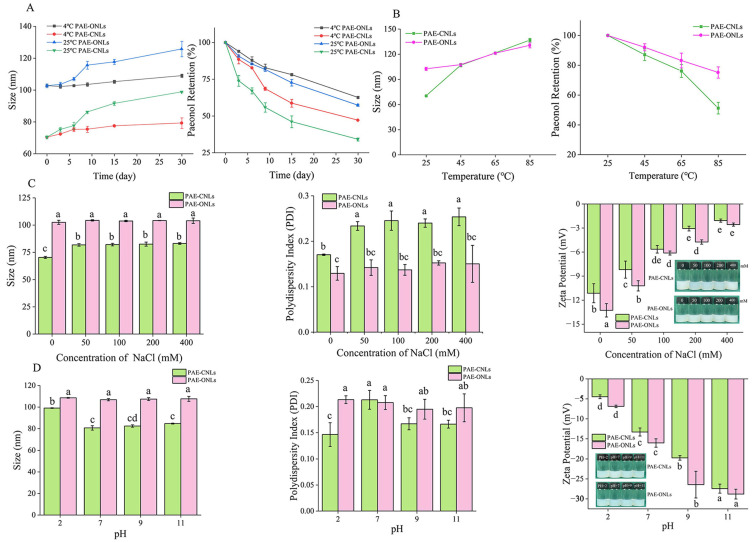
Stability studies of PAE-ONLs and PAE-CNLs. (**A**) Effect of storage time and temperature on particle size and paeonol retention. (**B**) Effect of temperature on particle size and paeonol retention. (**C**) Effect of ionic strength on particle size, polydispersity index, zeta potential, and appearance. (**D**) Effect of pH on particle size, PDI, zeta potential, and appearance. Data are presented as mean ± standard deviation (*n* = 3), with different letters indicating significant differences (*p* < 0.05) across samples.

The storage stability studies showed that PAE-ONLs maintained better size stability than PAE-CNLs over 30 days (Figure 4A). At 4 °C, PAE-ONLs showed a minimal size increase (from 102.57 nm to 108.97 nm) compared to PAE-CNLs (from 70.32 nm to 79.20 nm). Similarly, PAE-ONLs demonstrated superior paeonol retention (62.61%) at 4 °C compared to PAE-CNLs (57.31%). Storage at 25 °C resulted in lower retention rates of 47.1% and 34.07% for PAE-ONLs and PAE-CNLs, respectively.

#### 3.4.2. Thermal Stability

Under thermal stress (Figure 4B), PAE-ONLs exhibited better stability with only a 27.30% size increase at 85 °C, while PAE-CNLs showed a 94.44% increase. More importantly, PAE-ONLs maintained higher paeonol retention (75.15%) compared to PAE-CNLs (51.24%) at 85 °C, indicating superior thermal stability.

#### 3.4.3. Salt Stability

In the salt stability tests (Figure 4C), the PAE-ONLs’ size remained relatively stable throughout the NaCl concentrations (0–400 mM), whereas the PAE-CNLs’ size significantly increased (70.31 to 83.14 nm). Both formulations exhibited decreased zeta potentials with an increasing ionic strength, though their PDI values remained below 0.3, indicating sustained homogeneity.

#### 3.4.4. pH Stability

The pH stability studies (Figure 4D) revealed that PAE-ONLs remained constant in size across pH 2–11, although PAE-CNLs significantly increased in size at pH 2 (99.11 nm). Under alkaline conditions, the absolute zeta potential values of both formulations were at their maximum; PAE-ONLs showed lower negative values than PAE-CNLs (−27.43 and −28.80 mV).

In conclusion, this study found that the zeta potential of −13.3 mV, consistent particle size, and PDI provide moderate stability in colloidal materials. However, other factors like pH, particle size, ionic strength, stabilizing agents, and formulation conditions also contribute to the stability profile, highlighting the multifactorial nature of formulation stability.

### 3.5. Comparison of Thermodynamic Stability and Hydrogen Bonding

**Figure 5:**
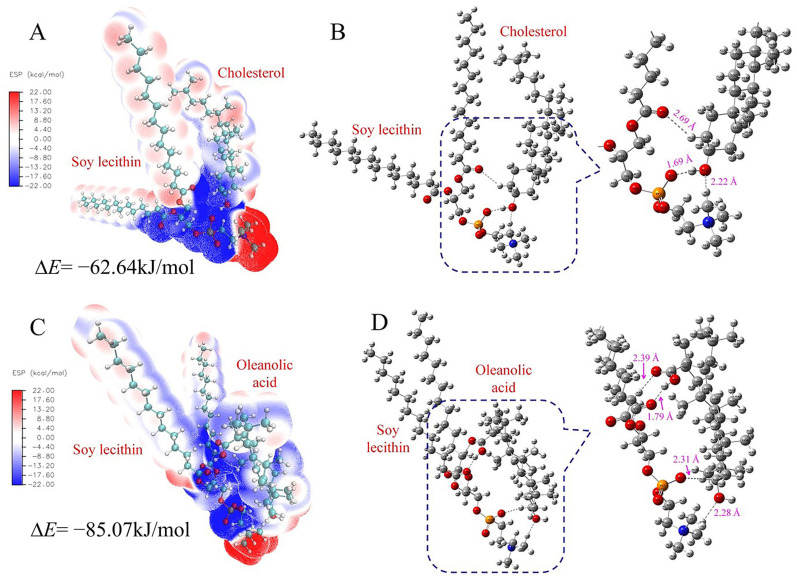
Molecular interaction between soy lecithin and cholesterol/oleanolic acid. (**A**) Electrostatic potential surface of soy lecithin and cholesterol. (**B**) Detailed interaction and bond lengths between soy lecithin and cholesterol. (**C**) Electrostatic potential surface of soy lecithin and oleanolic acid. (**D**) Detailed interaction and bond lengths between soy lecithin and oleanolic acid.

Based on Figure 5, DFT calculations revealed distinct molecular interactions and stability differences between the cholesterol and oleanolic acid-based liposomes. Electrostatic potential surface maps (Figure 5A,C) showed different charge distributions, while the detailed molecular interactions (Figure 5B,D) demonstrated that ONLs formed stronger and more numerous hydrogen bonds compared to CNLs. Specifically, CNLs showed hydrogen bond lengths varying from 1.69 Å to 2.69 Å, with a less favorable total energy decrease of −62.64 kJ/mol. In contrast, ONLs exhibited more stable hydrogen bonding interactions, with bond lengths ranging from 1.79 Å to 2.39 Å, and they achieved a greater energy reduction of −85.07 kJ/mol. The molecular interaction diagrams clearly show that oleanolic acid formed multiple hydrogen bonds with soy lecithin (bond lengths: 2.11 Å, 2.28 Å, and 2.39 Å), while cholesterol demonstrated fewer and weaker interactions (bond length: 2.22 Å), indicating that oleanolic acid substitution enhances the overall thermodynamic stability of the liposomal structure through stronger intermolecular bonding.

In conclusion, this study explored H-bond formation sites in liposomes and paeonol, focusing on C–O, O–H, and P–O groups. Multiple initial structures were designed and optimized using Gaussian16 software, with two being the most stable configurations. The dominant conformations fluctuated around these key sites. The electrostatic potential (ESP) analysis showed that H-bonds tended to form in the direction of the negative gradient. This study’s main goal was to understand the thermodynamic stability of the interactions between liposomes and paeonol. The results showed that the energy reduction in the system primarily came from hydrogen bonding contributions, indicating that such structures are feasible and can exist in macroscopic systems.

### 3.6. Evaluation of Anti-Inflammatory Activity in Zebrafish

**Figure 6:**
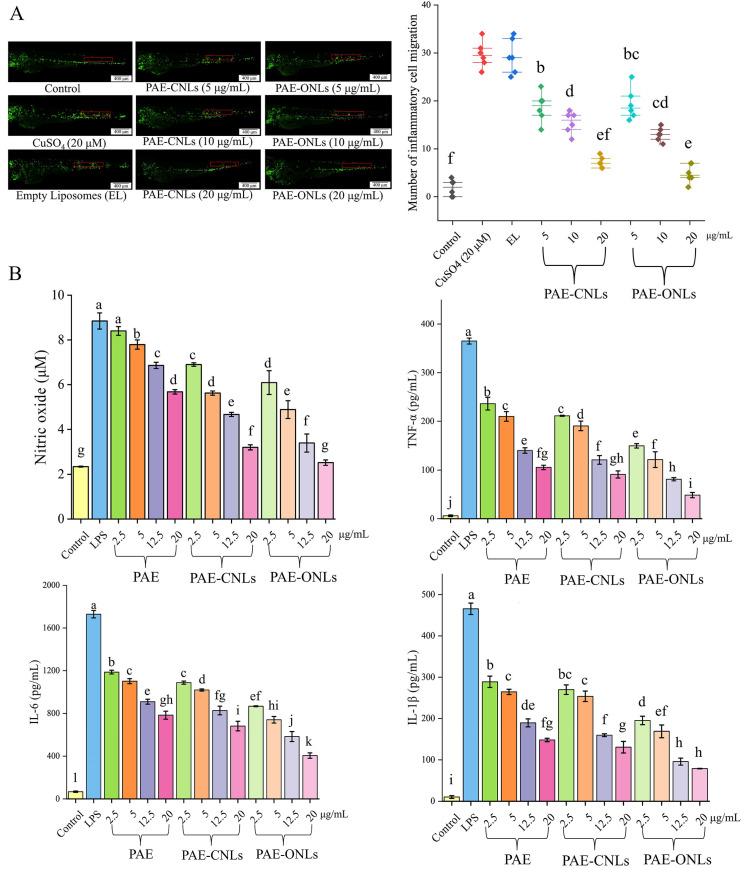
Anti-inflammatory effects of PAE-ONLs and PAE-CNLs. (**A**) Inhibition of inflammation in transgenic fluorescent zebrafish *Tg* (*Lyz: EGFP JS7*) in vivo. Significant differences (*p* < 0.05) across samples are represented by various letters, and the data reflect mean ± standard deviation (*n* = 6). (**B**) Pro- and anti-inflammation of different treatments in LPS-stimulated RAW 264.7 cells. Levels of NO, TNF-α, IL-6, and IL-1β in the culture supernatants were analyzed. The various letters indicate significant differences (*p* < 0.05) across samples, and the data show mean ± SD (*n* = 3).

Based on the zebrafish anti-inflammatory study results shown in Figure 6A, it was found that CuSO_4_ (20 μM) exposure significantly increased inflammatory cell migration to the lateral line compared to the control group, as evidenced by the intense green fluorescent signals along the lateral line in the CuSO_4_ model group image. Empty liposomes (40 μg/mL) had minimal effects on the inflammatory response, as indicated by similar fluorescence patterns to the CuSO_4_ group. Both PAE-CNLs and PAE-ONLs demonstrated concentration-dependent anti-inflammatory effects at 5, 10, and 20 μg/mL, visualized by progressively reducing green fluorescent signals in the microscopy images, with the most significant reduction in inflammatory cell migration observed at 20 μg/mL. The fluorescence imaging clearly shows that PAE-ONLs exhibited superior anti-inflammatory efficacy, with a 32.56% greater reduction in inflammatory cell migration compared to PAE-CNLs at the same concentration, as evidenced by notably weaker fluorescent signals in the PAE-ONL-treated groups. The corresponding quantitative analysis in the scatter plot confirms this observation, showing significantly lower inflammatory cell counts in the PAE-ONL groups compared to in PAE-CNLs at all tested concentrations. This visual and quantitative evidence strongly indicates that substituting cholesterol with oleanolic acid not only maintains but also improves the anti-inflammatory properties of the liposomal formulation.

### 3.7. Inflammation in RAW 264.7 Macrophages Stimulated by LPS

#### 3.7.1. Measurement of Cell Viability

The effects of PAE, PAE-CNLs, and PAE-ONLs on cell survival following a 24 h interaction with RAW 264.7 were examined in this work. As shown in Figure S2, the safe dosage range of the three experimental groups on RAW 264.7 cells was established with the CCK_8_ approach. When the concentration of the samples was 25 μg/mL, cell viability in the three experimental groups was comparable to or more than 80%. As a result, we chose to separate the experimental groups’ concentrations into 2.5, 5, 10, and 20 μg/mL for further testing.

#### 3.7.2. PAE-ONLs Inhibit Inflammatory Factors in LPS-Stimulated RAW 264.7 Macrophages

The anti-inflammatory effects of PAE, PAE-CNLs, and PAE-ONLs were evaluated by examining the inhibition of key inflammatory mediators in LPS-stimulated RAW 264.7 cells. As shown in Figure 6B, PAE-ONLs exhibited the strongest inhibition of NO production at 20 μg/mL, achieving a 71.50% reduction, which was superior to that exhibited by PAE-CNLs (63.70%) and PAE (35.80%). Similarly, PAE-ONLs demonstrated the most potent inhibition of TNF-α (86.60%), IL-6 (77.20%), and IL-1β (82.50%) production at 20 μg/mL, significantly outperforming both PAE-CNLs (75.10%, 61.50%, and 71.70%, respectively) and PAE alone (71.10%, 55.70%, and 68.30%, respectively).

### 3.8. Network Pharmacology-Based Investigation of the Synergistic Anti-Inflammatory Effects

**Figure 7:**
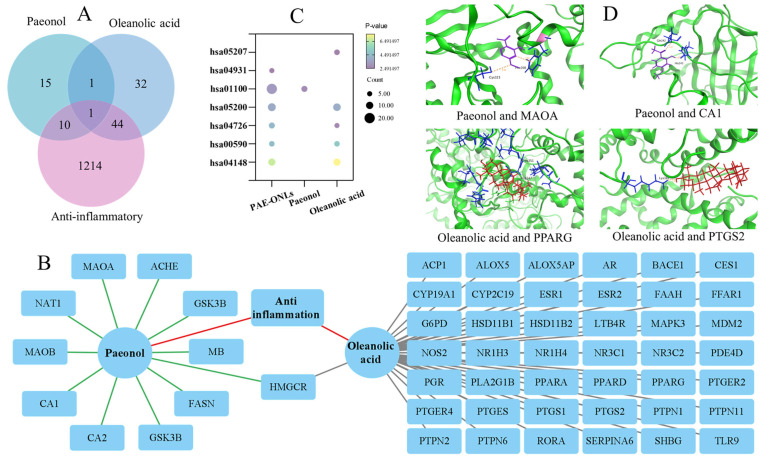
Network pharmacology analysis of anti-inflammatory targets and pathways of paeonol, oleanolic acid, and PAE-ONLs. (**A**) Venn diagram of anti-inflammatory targets. (**B**) Compound–Target–Disease network. (**C**) KEGG pathway enrichment analysis. (**D**) Molecular docking results.

A network pharmacology analysis revealed comprehensive target profiles for the investigated compounds. As shown in Figure 7A, the Venn diagram analysis demonstrated that paeonol interacted with 27 targets overall, with 11 specifically related to anti-inflammatory effects. Oleanolic acid showed a broader targeting capacity with 78 total targets, of which 45 were anti-inflammatory-related. Notably, their combination in PAE-ONLs expanded the coverage to 55 anti-inflammatory targets, suggesting potential synergistic effects in modulating inflammatory responses through multiple pathways.

The Compound–Target–Disease network analysis (Figure 7B) revealed intricate interactions between the compounds and their targets. The network visualization clearly distinguished the connections through color coding, with paeonol–target interactions in green, oleanolic acid–target interactions in gray, and anti-inflammatory connections in red. Notably, HMGCR was shown to be a target that was shared by oleanolic acid, paeonol, and anti-inflammatory activity, indicating that it may play a crucial role in mediating their therapeutic actions.

The KEGG pathway enrichment analysis (Figure 7C) identified seven critical inflammation-related pathways. These included chemical carcinogenesis—receptor activation, insulin resistance, metabolic pathways, pathways in cancer, serotonergic synapse, arachidonic acid metabolism, and efferocytosis. The diversity of these pathways indicates that PAE-ONLs can simultaneously regulate multiple aspects of inflammation and related biological processes.

Molecular docking studies (Figure 7D) provided detailed insights into the binding mechanisms of these compounds. The analysis demonstrated stable molecular interactions between paeonol and its targets (MAOA and CA1), as well as between oleanolic acid and its targets (PPARG and PTGS2). The binding conformations were visualized, with paeonol represented in purple, oleanolic acid in red, and target protein binding sites in blue, with interactions shown through dashed lines.

Further quantitative analysis of binding energies (Table S4) revealed strong interactions for both compounds. Paeonol showed particularly strong binding with MAOA (−5.4758 kcal/mol) and CA1 (−4.9090 kcal/mol), while oleanolic acid demonstrated robust interactions with PPARG (−6.2424 kcal/mol) and PTGS2 (−5.9657 kcal/mol). These binding energies suggest stable and specific interactions between the compounds and their respective targets, supporting their potential therapeutic efficacy in inflammatory conditions.

## 4. Discussion

Liposomes are widely used as drug delivery systems due to their excellent biocompatibility and ability to encapsulate both hydrophobic and hydrophilic bioactive substances. As a critical component of liposomes, cholesterol plays an important role in membrane stability but may pose health risks like hyperlipidemia and cardiovascular disease when consumed in excess [32]. Therefore, finding suitable alternatives to cholesterol while maintaining liposome stability is crucial. Hong et al. [33] demonstrated that ginsenoside rh2 can effectively replace cholesterol in paclitaxel liposomes to stabilize the structure of liposomes and prolong liposome circulation, and it also synergistically enhances the efficacy of anticancer drugs as an active ingredient. This structural similarity suggests that oleanolic acid can potentially replace cholesterol in liposome formulations. Additionally, oleanolic acid possesses various pharmacological properties, including antiviral, antitumor, anti-inflammatory, and anticancer effects [34,35,36,37], which can provide additional therapeutic benefits when incorporated into liposomal drug delivery systems. In this study, we successfully developed a novel liposome formulation (PAE-ONLs) using oleanolic acid instead of cholesterol to encapsulate paeonol, aiming to enhance both stability and therapeutic efficacy.

Single-factor experiments and response surface methodology (RSM) are important optimization approaches in pharmaceutical formulation. Single-factor experiments examine the effect of individual variables while keeping others constant, while RSM employs statistical designs to analyze multiple variables simultaneously and establish mathematical optimization models. These methods have been widely applied in liposome formulation optimization [38]. Wu et al. [27] used single-factor experiments and RSM to optimize lysozyme-loaded liposomes, achieving 85.6% encapsulation efficiency under their optimal conditions. Liu et al. [39] employed this approach for *p*-coumaric acid-loaded nanoliposomes, obtaining a 55.7% encapsulation efficiency through parameter optimization. In the present study, we utilized single-factor experiments and RSM to optimize PAE-ONL preparation, achieving a 64.61% encapsulation efficiency under optimal conditions. While this efficiency was relatively lower than that in previous reports, our study innovatively demonstrated the feasibility of using oleanolic acid as a cholesterol substitute in liposome formulation. The optimization process effectively improved preparation reproducibility while reducing material consumption [40].

Compared to traditional liposome preparation methods, microfluidic technology offers significant advantages, including better control over particle size, improved batch-to-batch consistency, and enhanced manufacturing efficiency [41]. Shah et al. [21] demonstrated that microfluidic-based liposome preparation could achieve a more uniform size distribution and better reproducibility compared to conventional bulk methods. In this work, we successfully prepared PAE-ONLs using microfluidic technology with optimized parameters (soy lecithin at 10.25 mg/mL, oleanolic acid at 0.82 mg/mL, and Tween 80 at 0.22%), achieving an encapsulation efficiency of 64.61 ± 0.42%, with an average particle size of 102.65 ± 2.55 nm and a PDI of 0.129 ± 0.015. The FTIR showed that oleanolic acid was effectively coupled to the liposomes, while the TEM investigation showed that the liposomes were uniform and spherical. These characterization results are consistent with previous findings by Shishir et al. [42] on pectin–chitosan-coated liposomes and demonstrate that improved uniformity and structural integrity were obtained through our microfluidic approach.

The stability of liposomes is a critical parameter that determines their therapeutic efficacy, storage conditions, and potential clinical applications. Yang et al. [43] enhanced the physicochemical stability and oral bioavailability of α-linolenic acid (ALA) by encapsulating it in nanoliposomes and decorating their surface with carboxymethyl chitosan (CMCS). DFT calculations provide theoretical insights into molecular interactions and structural stability at the quantum mechanical level. A previous study by Tian et al. [44] used DFT to investigate the interaction between phospholipids and paeonol in paeonol–liposomes and its effect on membrane stability, focusing primarily on binding energies. In our comprehensive stability assessment, PAE-ONLs demonstrated superior performance across multiple parameters, including storage stability (4 °C and 25 °C over 30 days), thermal stability (up to 85 °C), salt stability (up to 400 mM NaCl), and pH stability (pH 2–11). Furthermore, our DFT calculations revealed that PAE-ONLs possessed a larger energy reduction (−85.07 kJ/mol) and stronger hydrogen bonding compared to traditional cholesterol-based liposomes. These experimental findings align well with our theoretical predictions and previous studies by Han et al. [12] while extending beyond their initial observations to demonstrate enhanced stability across a broader range of conditions.

Inflammation is a complex biological response characterized by the production of pro-inflammatory mediators, including TNF-α, NO, IL-6, and IL-1β. In earlier research, Iqbal et al. [45] used a zebrafish model to evaluate the cardiotoxicity of paeonol by activating Notch1 signaling and measuring the expression of its downstream target genes. The anti-inflammatory properties of oleanolic acid were examined by Lee et al. [46] in RAW 264.7 macrophages by enhancing barrier integrity, suppressing CAM expression, and preventing neutrophil adhesion and TEM across endothelial cells. In our study, zebrafish experiments revealed that PAE-ONLs significantly inhibited inflammatory cell migration to the lateral line, showing a 32.6% greater inhibition compared to PAE-CNLs. In RAW 264.7 cell experiments at 20 μg/mL, PAE-ONLs demonstrated superior inhibition rates of 71.50%, 86.60%, 77.20%, and 82.50% for NO, TNF-α, IL-6, and IL-1β, respectively, significantly outperforming PAE-CNLs (63.70%, 75.10%, 61.50%, and 71.70%). The results of both models consistently demonstrated that PAE-ONLs possessed superior anti-inflammatory activity compared to traditional PAE-CNLs. This enhanced efficacy can be attributed to both improved bioavailability and stability, as well as the synergistic effects of the combined compounds, which are consistent with the synergistic antitumor results of sea cucumber saponins replacing adriamycin cholesterol in liposomes by Zhang et al. [47].

Network pharmacology represents a novel approach to understanding drug mechanisms of action by analyzing the complex interactions between drugs and biological systems at a molecular level [48]. This methodology integrates systems biology, network analysis, and pharmacology to elucidate the multi-target effects of therapeutic compounds. Previous studies have employed network pharmacology to investigate traditional anti-inflammatory compounds. For instance, Chen et al. [13] found that paeonol targets multiple inflammatory pathways, particularly those involving NF-κB signaling. Similarly, Shim et al. [49] demonstrated that oleanolic acid modulates various inflammation-related targets through network pharmacology analyses. In our study, we utilized network pharmacology to comprehensively analyze the molecular mechanisms of PAE-ONLs. The results revealed a complex interaction network, where paeonol interacts with 11 anti-inflammatory targets, while oleanolic acid affects 45 targets. Notably, their combination in PAE-ONLs demonstrated an expanded influence on 55 anti-inflammatory targets, indicating significant synergistic effects. The Compound–Target–Disease network analysis identified HMGCR as a crucial common target for both compounds, suggesting a mechanistic basis for their synergistic action. Similar to the findings of Wu et al. [50], a KEGG pathway enrichment analysis revealed that PAE-ONLs influence multiple inflammation-related pathways simultaneously, including arachidonic acid metabolism and insulin resistance pathways. Molecular docking studies complemented these findings, demonstrating stable binding interactions of both compounds with key inflammatory targets, such as MAOA, CA1, PPARG, and PTGS2. The consistency between these network pharmacology findings and experimental results in both zebrafish and RAW 264.7 cell models validates the computational predictions, demonstrating the reliability of network pharmacology in understanding complex drug mechanisms. In conclusion, this work focuses on the physicochemical properties and anti-inflammatory effect of PAE-ONLs in vitro and in zebrafish models. The formulation’s nanoscale size, stability, and biocompatibility make it an attractive choice for injectable, targeted, or systemic anti-inflammatory treatment. It might potentially be made into topical treatments for localized skin irritation. The liposomal method might also be modified for oral administration to increase bioavailability and preserve active chemicals from gastrointestinal breakdown.

## 5. Conclusions

In this work, PAE-ONLs were successfully prepared using microfluidic technology, with optimized conditions of soy lecithin (10.25 mg/mL), oleanolic acid (0.82 mg/mL), and Tween 80 (0.22%). The formulation exhibited uniform characteristics with an average particle size of 102.65 nm, a PDI of 0.129, and an encapsulation efficiency of 64.61%. TEM imaging confirmed the spherical morphology and uniform dispersion of PAE-ONLs, while FTIR spectroscopy verified the successful incorporation of oleanolic acid into the liposomal structure. PAE-ONLs demonstrated superior stability during storage and under various thermal, pH, and ionic conditions compared to traditional PAE-CNLs. This enhanced stability was supported by DFT calculations, which revealed stronger molecular interactions in PAE-ONLs through a greater energy reduction (−85.07 kJ/mol) and more stable hydrogen bonding (1.79–2.39 Å) compared to PAE-CNLs (−62.64 kJ/mol, 1.69–2.69 Å). The superior anti-inflammatory efficacy of PAE-ONLs was demonstrated in both zebrafish models and RAW 264.7 macrophages, showing a significantly higher inhibition of inflammatory mediators than PAE-CNLs. This enhanced therapeutic effect was mechanistically explained through a network pharmacology analysis, which revealed that paeonol and oleanolic acid individually affected 11 and 45 anti-inflammatory targets, respectively, and their combination in PAE-ONLs expanded the coverage to 55 targets through multiple pathways, confirming their synergistic therapeutic potential. These findings suggest that PAE-ONLs represent a promising strategy for clinical anti-inflammatory applications. In conclusion, this study provides valuable preliminary data for OA replacement liposomes for cholesterol, but in-depth exploration is still needed in terms of in vivo efficacy/toxicity, mechanism validation, and clinical translational feasibility. Future studies could incorporate more complex animal models, an experimental validation of targets, the optimization of encapsulation rates, and an assessment of scale-up production potential to advance practical applications.

## Data Availability

The original contributions presented in this study are included in the article/Supplementary Materials. Further inquiries can be directed to the corresponding author.

## Supplementary Materials

The following supporting information can be downloaded at: https://www.mdpi.com/article/10.3390/foods14122030/s1, Figure S1: Standard curve of paeonol; Figure S2: Effects of PAE, PAE-CNLs, and PAE-ONLs on the cell viability of RAW 264.7 cells; Table S1: Levels and code of variables chosen for Box−Behnken design; Table S2: Box−Behnken design and results; Table S3: ANOVA of response surface quadratic model; Table S4: Molecular docking binding energy of paeonol and oleanolic acid with hub targets.

## Abbreviations

PAE: paeonol; OA, oleanolic acid; PAE-ONLs, paeonol–oleanolic acid liposomes; PAE-CNLs, paeonol–cholesterol liposomes; ONLs, oleanolic acid liposomes; CNLs, cholesterol liposomes; RSM, response surface methodology; PDI, polydispersity index; TEM, transmission electron microscopy; FTIR, Fourier transform infrared spectroscopy; *EE*, encapsulation efficiency; DFT, density functional theory; HPLC, high-performance liquid chromatography; BBD, Box–Behnken design; DLS, dynamic light scattering; d-H_2_O, deionized water; CCK_8_, Cell Counting Kit-8; NO, nitric oxide; IL-6, interleukin-6; TNF-α, tumor necrosis factor-alpha; LPS, lipopolysaccharide; IL-1β, interleukin-1 beta; DMEM, Dulbecco’s Modified Eagle Medium; FBS, fetal bovine serum; ELISA, enzyme-linked immunosorbent assay.
