# Supplementary material for: Study of the Stability and Anti-Inflammatory Activity of Paeonol–Oleanolic Acid Liposomes by Microfluidic Technology
Source: Foods. 2025 Jun 8;14(12):2030. doi: 10.3390/foods14122030 (PMC12191921; doi:10.3390/foods14122030)
Supplement: Supplementary file 1 [file foods-14-02030-s001.zip › foods-3674968-supplementary.pdf]

Article

Study of the Stability and Anti-Inflammatory Activity of Paeonol–Oleanolic Acid Liposomes by Microfluidic Technology

Xianzheng Ma [1], Hui Zhang [1], Jinkai Luan [1], Mingfa Tian [1], Xiuxin Zhang [2], Ammara Sohail [1,3], Dong Liang [4], Jiguo Liu [4], Fuzhan Tao [4], Zheng Wang [5] and Daijie Wang [1,*]

1 College of Pharmacy, Food Resources Development and Health Product Creation International Joint Laboratory/Biological Engineering Technology Innovation Center of Shandong Province, Heze Branch of Qilu University of Technology, Shandong Academy of Sciences, Heze 274000, China; 15106912350@163.com (X.M.); zhcharmingqueen@163.com (H.Z.); kari323682567@163.com (J.L.); t15165141920@163.com (M.T.); sohailammara9@gmail.com (A.S.)

2 Institute of Vegetables and Flowers, Chinese Academy of Agricultural Science, Beijing 100081, China; zhangxiuxin@caas.cn

3 Department of Chemistry, University of Okara, Okara 56300, Pakistan

4 Heze Peony Industrial Technology Research Institute, Heze 274000, China; szfbgsmdb@163.com (D.L.); hzljg919@163.com (J.L.); hzstfz@126.com (F.T.)

5 Department of Genetics and Cell Biology, Basic Medical College, Qingdao University, Qingdao 266071, China; zheng.wang@qdu.edu.cn

* Correspondence: wangdaijie@qlu.edu.cn or wangdaijie@126.com

Supplementary Tables

Table S1 Levels and code of variables chosen for Box–Behnken Design

Factors	code	Level and range		
		-1	0	1
Soy lecithin concentration (mg/mL)	A	8	10	12
Oleanolic acid concentration (mg/mL)	B	0.6	0.8	1.0
Tween 80 concentration (wt%)	C	0.15	0.2	0.25

Table S2 Box–Behnken design and results

Run	Variable levels			Responses
	A	B	C	*EE* (%)
1	12	1.0	0.20	55.78
2	10	0.8	0.20	64.14
3	10	0.6	0.15	57.62
4	8	1.0	0.20	56.62
5	8	0.8	0.25	55.27
6	10	1.0	0.25	60.73
7	8	0.6	0.20	52.71
8	10	1.0	0.15	57.14
9	8	0.8	0.15	54.14
10	10	0.8	0.20	64.30
11	12	0.6	0.20	57.31
12	12	0.8	0.25	60.02
13	10	0.8	0.20	64.04
14	10	0.8	0.20	63.79
15	10	0.6	0.25	56.62
16	10	0.8	0.20	64.70
17	12	0.8	0.15	55.79

A, *B*, and *C* represent soy lecithin concentration (mg/mL), oleanolic acid concentration (mg/mL), and tween 80 concentration (wt%), respectively.

Table S3 ANOVA of response surface quadratic mode

**Source**	**Sum of Squares**	**Degree of freedom**	**Mean Squares**	***F*-value**	***p*-value***
Model	253.91	9	28.21	79.54	< 0.0001
A	12.9	1	12.9	36.38	0.0005
B	4.52	1	4.52	12.73	0.0091
C	7.9	1	7.9	22.27	0.0022
AB	7.4	1	7.4	20.86	0.0026
AC	2.4	1	2.4	6.77	0.0353
BC	5.27	1	5.27	14.85	0.0063
A^2	111.92	1	111.92	315.56	< 0.0001
B^2	49.63	1	49.63	139.93	< 0.0001
C^2	31.46	1	31.46	88.69	< 0.0001
Residual	2.48	7	0.3547		
Lack of Fit	2.03	3	0.6752	5.91	0.0595
Pure Error	0.4571	4	0.1143		
Cor Total	256.39	16			
$R^2 = 0.9903$	$R^2_{Adj} = 0.9779$	$R^2_{Pred} = 0.8708$			

*Significant if $p < 0.05$

Table S4 Molecular docking binding energy of paeonol and oleanolic acid with hub targets.

	**Binding Energy (kcal/mol)**				
	1	2	3	4	5
Paeonol	MAOA	CA1	CA2	GSK3B	ACHE
	-5.4758	-4.9090	-4.8408	-4.781	-3.9779
Oleanolic acid	PPARG	PTGS2	PPARA	ESR1	MAPK3
	-6.2424	-5.9657	-5.7067	-5.6853	-5.2564

Supplementary Figures

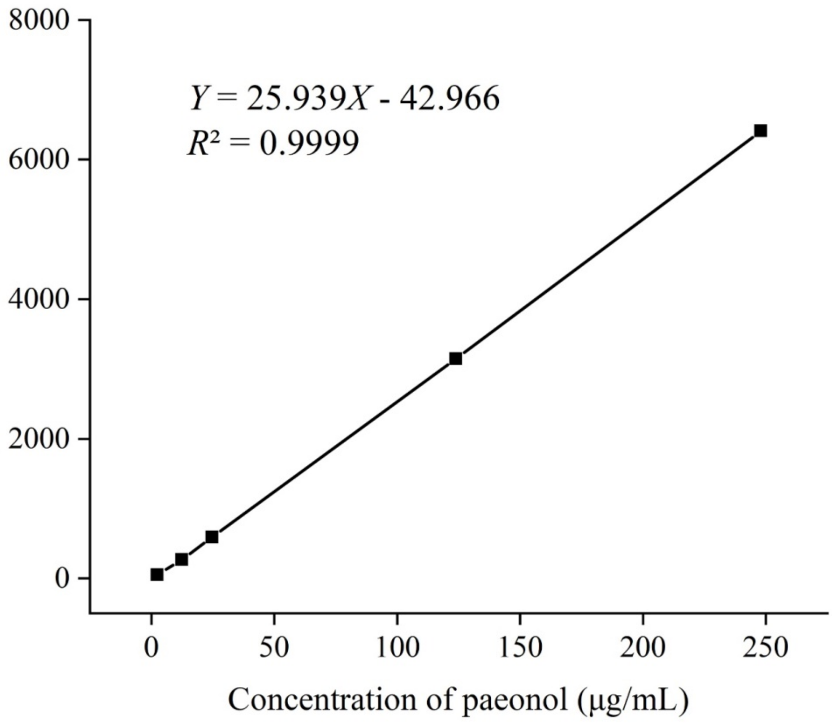

Figure S1 Standard curve of paeonol.

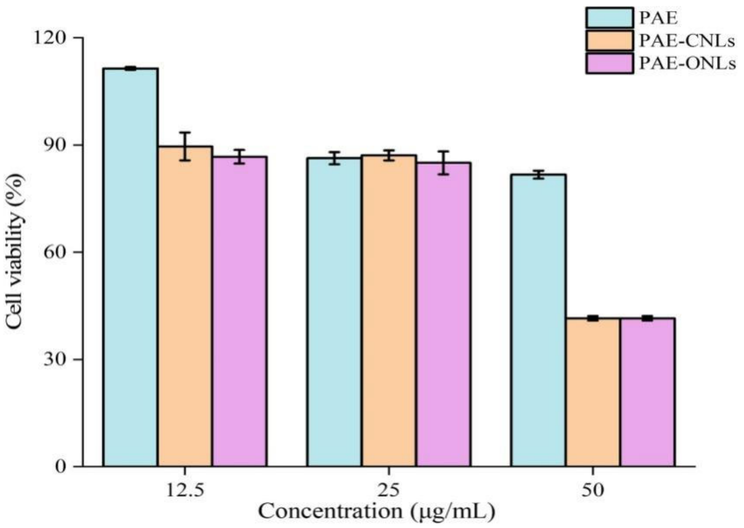

Figure S2 Effects of PAE, PAE-CNLs and PAE-ONLs on the cell viability of RAW264.7 cells.
